# Two Perspectives

**DOI:** 10.1007/s10912-023-09832-y

**Published:** 2023-12-16

**Authors:** Katarzyna Rakoczy

**Affiliations:** Faculty of Medicine, Wroclaw Medical University, Wroclaw, Poland

**Keywords:** Medicine, Life, Relationship, Conversation

It is not for us to greet each other or bid farewell we live on archipelagos

and that water these words what can they do what can they do prince

—Z. Herbert, “Elegy of Fortinbras”

## Chapter 1

if only I could voice

the beauty of being

with my own tears and smiles

I would declaim

the poetry of light

that opened my constantly closed eyes

the safety of your warmth

that made me feel so loved under your heart

the miracle of the tiny muscle’s very first beat

that gave my few weeks

their inimitable melody

lasting until

the last not-taken breath

though I could not enjoy the smell of the air

or experience the constraint of gravity

though I could not experience the tenderness of your touch

or even look into the world’s sky blue eyes

I am grateful for each second

for the mark that I left in your memory

after all today

it is the only thing that proves

that I really existed

and when

the blood was spinning the bright star in my chest

I was brave

I was brave Mommy

## Chapter 2

it must be stated that in fact nothing much happened

those who have to watch the world changing day by day

must be aware of their own transiency

whereas you are not obliged to fear the end

to experience the pressure whispering that

everything is going to collapse fade disappear

it must be stated that in fact nothing wrong happened

let’s call the things by name it was the act of sympathy

I spared you those burdens

loneliness longing

tones of divorce papers and

anti-aging creams that never actually work

I spared you escaping from indispensability

it must be stated that in fact nothing noteworthy happened

you would live-disappear invisible-unnoticed

forcibly returning your body to this world which

will never give anything to anyone

in non-superficial possession

and you could quit your frail circle of life even before you

became pathetically aware of the absurdity of circulation

when I saw you for the first and the last time I thought

that maybe you could have

enjoyed those few for evermore meaningless

fractions of eternity

the sunrise the sunset walk in the rain the piece of cake

the good word the gesture the glance the smile

but those words of my penance

what can they do what can they do.

## Data Availability

Data available within the article or its supplementary materials.

